# Immunocytes interact directly with cancer cells in the tumor microenvironment: one coin with two sides and future perspectives

**DOI:** 10.3389/fimmu.2024.1388176

**Published:** 2024-05-22

**Authors:** Zhiyi Ye, Pu Cheng, Qi Huang, Jingjing Hu, Liming Huang, Guoming Hu

**Affiliations:** ^1^ Department of General Surgery (Breast and Thyroid Surgery), Shaoxing People’s Hospital; Shaoxing Hospital, Zhejiang University School of Medicine, Zhejiang, China; ^2^ Department of Gynecology, The Second Affiliated Hospital of Zhejiang University School of Medicine, Hangzhou, China; ^3^ Department of Oncology, The Second Affiliated Hospital of Anhui Medical University, Hefei, Anhui, China; ^4^ Department of Oncology, Anhui Medical University, Hefei, Anhui, China; ^5^ School of Medicine, Shaoxing University, Zhejiang, China; ^6^ Department of General Surgery (Breast and Thyroid Surgery), Shaoxing People’s Hospital, Shaoxing Hospital, Zhejiang University School of Medicine, Shaoxing, Zhejiang, China; ^7^ Key Laboratory of Cancer Prevention and Intervention, Ministry of Education, Hangzhou, Zhejiang, China

**Keywords:** immunocytes, cancer cells, direct and dual effect, tumor microenvironment, solid tumor

## Abstract

The tumor microenvironment is closely linked to the initiation, promotion, and progression of solid tumors. Among its constitutions, immunologic cells emerge as critical players, facilitating immune evasion and tumor progression. Apart from their indirect impact on anti-tumor immunity, immunocytes directly influence neoplastic cells, either bolstering or impeding tumor advancement. However, current therapeutic modalities aimed at alleviating immunosuppression from regulatory cells on effector immune cell populations may not consistently yield satisfactory results in various solid tumors, such as breast carcinoma, colorectal cancer, etc. Therefore, this review outlines and summarizes the direct, dualistic effects of immunocytes such as T cells, innate lymphoid cells, B cells, eosinophils, and tumor-associated macrophages on tumor cells within the tumor microenvironment. The review also delves into the underlying mechanisms involved and presents the outcomes of clinical trials based on these direct effects, aiming to propose innovative and efficacious therapeutic strategies for addressing solid tumors.

## 1 Introduction

The tumor microenvironment (TME), consisting of immunocytes, stromal cells, extracellular matrix (ECM), and blood and lymphatic vascular networks, forms a complex immunomodulatory network (1, 2). In recent years, attention has been focused on understanding how immune cells, stromal cells, and cytokines regulate tumor cell proliferation, growth, metastasis, and invasion within the TME (3–5). Rather than functioning in isolation, these components of the TME synergistically interact to form an integrated entity (1). What’s more, disruptions in any part of this network may significantly impact overall tumor behavior. Therefore, a comprehensive grasp of the intricate dynamics inherent in the TME is imperative for forming efficacious cancer therapies.

Even more noteworthy is that immune cells, as a critical component in the TME, significantly contribute to maintaining human health. They play a pivotal role in recognizing, targeting, and eliminating mutated cells within the body (6, 7). This function is not only achieved through indirect pathways, such as adjusting the functionality and differentiation of other cells through the secretion of cytokines, but also through directly influencing the survival and subsequent progression of tumor cells (8). In contrast to the complexities inherent in indirect actions and the multifaceted interplay of reciprocal regulations, immunocytes’ direct cytotoxic effects offer a clear and unequivocal avenue for tumor treatment (9, 10). These direct interactions usually remain unaffected by intermediate multi-step modulations, resulting in potent cytotoxicity or significant direct promoting effects (11). However, this dual nature complicates immune therapy, closely tying it to the current challenge of achieving effective treatment for certain malignancy (12).

Therefore, we discuss the direct interplay between various immunocytes and neoplastic cells, coupled with an ensuing discourse on related treatments and clinical applications, alongside the extant obstacles, which may be beneficial for further research.

## 2 Direct cytotoxic effects of immune cells and their counteractions

### 2.1 T cells

#### 2.1.1 CD4^+^ T cells

The differentiation process of CD4^+^ T cells is governed by multiple factors, including antigen-specific stimulation, T cell receptors(TCR), cytokines, and transcription factors (13). Initially, upon detection of “non-self” or foreign substances by the immune system, antigens are presented to CD4^+^ T cells via the TCR, initiating the differentiation process (14). The type of antigen presenting cell(APC) determines the antigen type, while its affinity and quantity influence the nature and strength of TCR signaling, collectively regulating the activation and differentiation of CD4^+^ T cells in conjunction with co-stimulatory molecules (13, 14).

Subsequently, cytokines secreted by antigen-presenting cells and differentiated CD4^+^ T cells play crucial roles in differentiation. For instance, interleukin-12(IL-12) and interferon-gamma(IFN-γ) promote type 1helper T(Th1) cell differentiation (15, 16), IL-4 induces Th2 cell differentiation (17), IL-4 and transforming growth factor beta(TGF-β) enhance Th9 cell differentiation (18, 19), and IL-6 and TGF-β drive Th17 cell differentiation (20). These cytokines activate distinct signaling pathways, guiding the formation of specific T cell subgroups (13).

Finally, under the regulation of specific cytokine signals and cellular environments, master transcription factors contribute to shaping and maintaining the balance and diversity of the immune system by activating specific gene expression patterns (16). Each T cell subset is governed by lineage-specific master transcription factors, such as T-bet, GATA binding protein 3(GATA3), interferon regulatory factor 4(IRF4), and retinoid-related orphan receptor gamma t(RORγt), which control the expression of subset-specific genes, thereby determining the direction of cell differentiation (18, 21–24). Therefore, the variegated landscape of the tumor microenvironment impels T cells toward distinct subtypes, underscoring the critical importance of the types and functional states of T cell subtypes in shaping the immune response to tumors (13).

##### 2.1.1.1 Th1 cells

Historically, CD4^+^ T cells have been construed as orchestrators of immune responses, activating and recruiting other immune cells by producing their distinctive cytokines (25). In contrast, CD8^+^ T cells are intricately associated with the direct elimination of target cells (26). Nevertheless, recent years have witnessed an in-depth exploration of the intricacies of CD4^+^ T cell functionality, particularly those cells exhibiting antigen-specific cytotoxic activity, denoted as CD4^+^ cytotoxic T lymphocytes (CTLs) (27).

These CD4^+^ T lymphocytes have been demonstrated to elicit cytotoxic effects on tumor cells by directly releasing granule enzymes (28–31). Additionally, they have been validated to implement cytotoxic responses in solid tumors such as melanoma and lymphoma through mediation of the factor associated suicide/factor associated suicide ligand(Fas/FasL) and tumor necrosis associated apoptosis-inducing ligand(TRAIL) pathways (32–34). While in the initial phases, CD4^+^ CTLs were erroneously classified within the Th1 cell subset (25). However, lamentably, there is a dearth of conclusive evidence substantiating the assertion that Th1 cells can induce direct cytotoxicity against tumor cells through the three pathways above.

**Figure 1:**
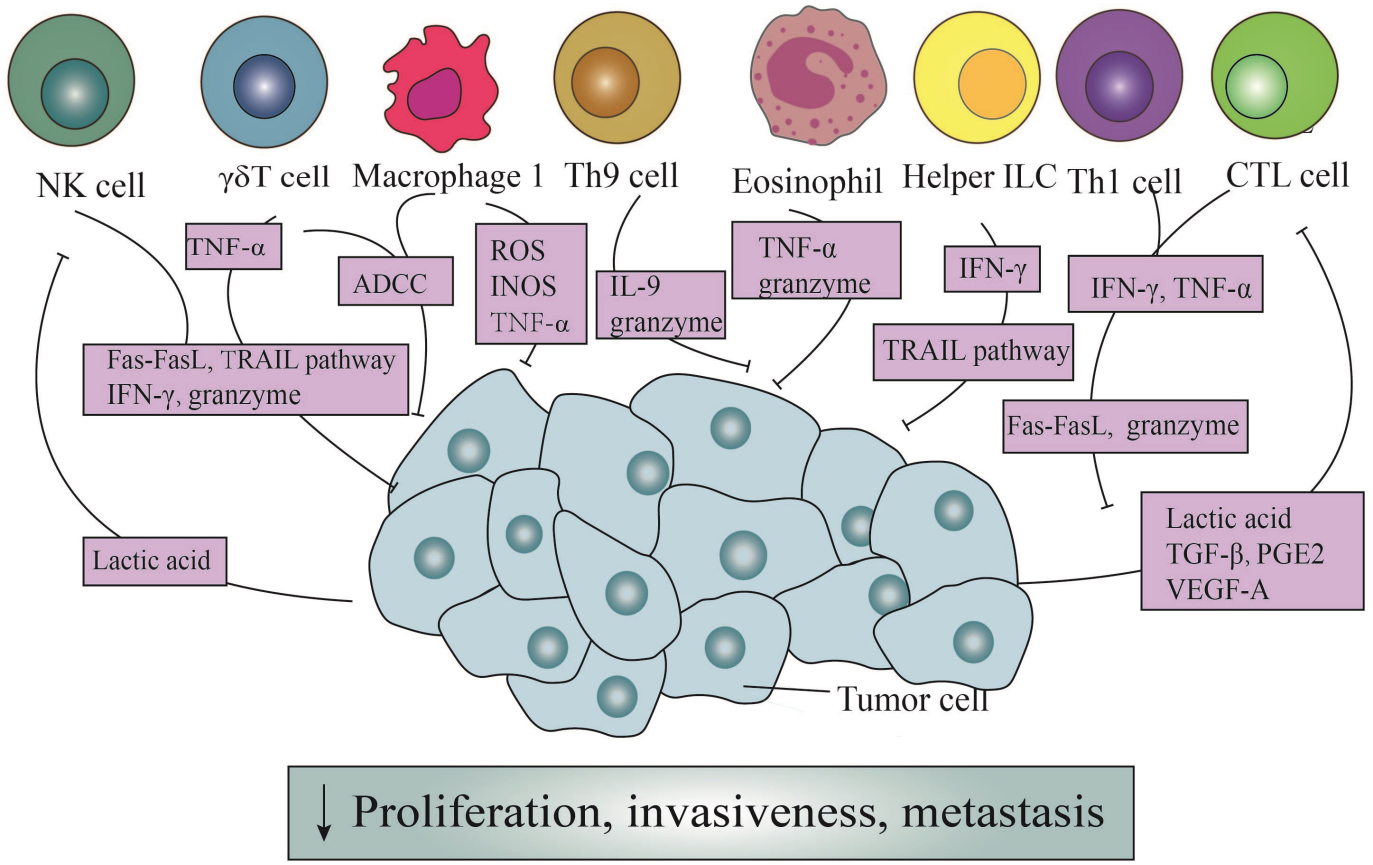
The direct antitumor action of immune cells and the counteraction of tumor cells. Through the Fas/Fasl pathway, ADDC pathway, and TRAIL pathway, immune cells exert direct cytotoxic effects on tumor cells. Simultaneously, they can release granule enzymes, IFN-γ, TNF-α, ROS, INOS, and other mediators to generate cytotoxicity. In addition, Th9 cells induce apoptosis in tumor cells by releasing IL-9. It is noteworthy that tumor cells, in turn, enhance the cytotoxicity of NK cells and CTL cells through secretion lactic acid, TGF-β, PGE2, and VEGF-A.

However, Th1 cells efficaciously manifest their anticancer prowess through the secretion of IFN-γ (**Figure 1**). Primarily, the IFN-γ orchestrates a reduction in the envelopment of peripheral tumor cells, facilitating the aberrant genesis of vasculature and the regularization of vascular architecture, thereby impeding the proliferation of tumor vasculature (35). These actions possess the potential to perturb the oxygenic and nutritive milieu within the TME, ultimately precipitating the demise of neoplastic cells (36). Additionally, the impact of IFN-γ extends through the orchestrated proteasomal degradation of the human epidermal growth factor receptor 2(HER2) membrane receptor, mediated by the E3 ubiquitin ligase cullin-5, inducing the senescence of tumor cells in breast cancer (37). According to the latest pancreatic cancer study, the collaboration of Th1 cell-derived IFN-γ with tumor necrosis factor(TNF) triggers a state of enduring growth arrest in the G1/G0 phase, activates p16 the inhibitor of cyclin-dependent kinase 4a(p16INK4a), and instigates downstream hypophosphorylation of the Rb protein at serine residues, thereby effectuating the senescence of β-pancreatic cancer cells (38) (**Figure 1**).

##### 2.1.1.2 Th9 cells

In the presence of IL-4 and TGF-β1, naïve CD4^+^ T cells exhibit the capacity to differentiate into a distinct subset known as Th9 cells (39). These cells possess the ability to generate IL-9, a cytokine initially proposed to be involved in promoting tumorigenesis (39). However, subsequent investigations have revealed its anti-tumor effects. Purwar et al. pioneered the demonstration of Th9 cells’ efficacy in suppressing melanoma growth upon injection into murine hosts, outperforming the effects of Th1, Th2, and Th17 cells (40). The heightened efficiency in inducing tumor cell apoptosis was intricately associated with the elevated expression of granzyme B, the blockade of which markedly mitigated the cytotoxic effects (23, 40) (**Figure 1**). Further elucidation of Th9 cells unveils their proficiency in eradicating advanced tumors. Studies emphasize Eomesodermin as a principal regulatory factor governing the expression of cytotoxic enzymes. Augmentation of Eomesodermin coincides with an increase in the gene expression of the cytotoxic enzyme repertoire within Th9 cells (41).

In addition, IL-9 is critical for suppressing tumor growth (42) (**Figure 1**). In murine melanoma experiments, the increase in both the quantity of Th9 cells and IL-9 significantly reduces the tumor growth rate, despite *in vitro* studies demonstrating a close association with their indirect effects (43). Another study centered around HTB-72 and SK-Mel-5 melanoma cells has established a link between the anti-proliferative effects of IL-9 and the heightened expression of p21 (44). Besides, a discernibly elevated count of apoptotic cells following IL-9 treatment has been observed compared to when contrasted with the control group, further validating their conclusion (44).

#### 2.1.2 CD8^+^ T cells

Under antigen stimulation, naïve CD8^+^ T cells generate effector and memory T cells, with the effector CD8^+^ T cells referred to as CD8^+^ CTLs (13). CTLs assume a pivotal role in the vigilant immune surveillance against neoplastic entities, recognizing cell surface antigens on tumor cells through the discerning receptors of the TCR (44, 45). The primary mechanisms through which CTLs coordinate their anti-tumor capabilities involve the granzyme/perforin pathway and cytotoxicity mediated by Fas receptors (46). The granzyme/perforin cascade involves the liberation of granules containing granzymes and perforin, thereby directly instigating apoptotic cascades within targeted cellular domains (47–49). Usually, perforin serves as the conduit for ingressing granzymes into tumor cells, thereby facilitating the demise of targeted cells (50). Therefore, the absence or impairment of perforin may diminish the tumor-suppressive efficacy of CTLs (47). In the latest literature, it has been discovered that endosomal sorting complexes required for transport can repair the plasma membrane pores caused by perforin. This rehabilitative action restores membrane integrity, effectively preventing the invasion of granzymes (51) (**Figure 1**).

In instances where the integrity of the granzyme/perforin pathway is compromised, there emerges a heightened prominence of Fas-mediated processes (46) (**Figure 1**). FasL triggers apoptosis through the intricate activation of caspases (52). Nonetheless, noteworthy observations posit that FasL expressed by exosomes might exert divergent effects, potentially fostering tumor invasion instead of inducing apoptotic signals (53).

Furthermore, CTLs also possess the capability to modulate the metabolic dynamics of neoplastic cells through the secretion of cytokines (**Figure 1**). Notably, factors such as IFN-γ, originating from CTLs, downregulate certain components of the glutamate-cystine antiporter system, subsequently influencing lipid metabolism within the tumor cell milieu and promoting tumor cells’ apoptosis (54, 55). Another secreted product, TNF-α, despite its potential derivation from various immune cells, undeniably plays a role in inducing the rupture of tumor blood vessels, promoting cell infiltration, and maintaining an ischemic state in tumors (56) (**Figure 1**). However, under typical circumstances, the contact of individual CTLs with tumor cells fails to eliminate the tumor cells effectively. And it is only through sequential interactions with multiple CTLs that elimination occurs (57).

In addition, tumor cells heavily impact the function of CTLs (**Figure 1**). Tumor-derived lactic acid efficaciously reduces the activity of monocarboxylate transporter -1, which weakens cellular metabolism and diminishes the cytotoxicity of IFN-γ, granzymes, and perforin in CTLs (58). Moreover, the secretion of TGF-β by tumor cells directly impedes the immune activity of CTLs by inducing the upregulation of miR-23a and simultaneous downregulation of B-lymphocyte-induced maturation protein 1(Blimp-1) (59). Notably, Blimp-1, as a pivotal transcriptional repressor, plays a fundamental role in the differentiation and memory response of effector CD8^+^ T cells (60). Consequently, this mechanism undermines the immune function mediated by CTLs. Tumor cells also induce the expression of FasL in endothelial cells via vascular endothelial growth factor-A (VEGF-A), IL-10, and prostaglandin E2 (PGE2), thereby eliciting specific cytotoxic effects in effector T cells (61).

#### 2.1.3 Gammadelta T cells (γδT cells)

γδT cells and αβT cells are the two main types of T cells within the adaptive immune system. γδT cells have T-cell receptors composed of γ and δ chains and recognize a broader range of antigens, while αβT cells bear T-cell receptors made of α and β chains and primarily respond to peptide antigens presented by major histocompatibility complex (MHC) molecules (62).

Despite being a minority among peripheral blood cells, γδT cells assume a pivotal role in the detection and eradication of tumor cells (63). In a previous study focused on squamous cell carcinoma of the head and neck (SCCHN), it was observed CD56^+^ γδT cells, isolated from peripheral blood mononuclear cells (PBMCs) expanded under the stimulation of isopentenyl pyrophosphate (IPP) and IL-2, could effectively destroy SCCHN cell lines in a dose-dependent manner, in contrast to CD56^-^ γδT cells (64). What’s more, the cytotoxicity of γδT cells underwent a notable suppression following treatment with concanamycin A (CMA), an inhibitor of the granzyme/perforin pathway (64), which concurrently functions as a downregulator (65). Additionally, γδT cells also exhibit a lytic effect on MCF-7 breast tumor cells. Subsequent research revealed that MCF-7 tumor cells were surrounded by a substantial number of γδT cells, forming a tight conjugate, and were subsequently eliminated within a span of ten seconds. Furthermore, γδT cells were empirically demonstrated to possess the capability to lyse autologous primary tumor kidney cells, a phenomenon alleviated upon the application of CMA (66). From the above, it can be deduced that the perforin/granzyme pathway occupies an irreplaceable position in the cytotoxic activity of γδT cells (67) (**Figure 1**).

Antibody-dependent cell-mediated cytotoxicity (ADCC) constitutes another crucial mechanism (68) (**Figure 1**). Classified by their maturation levels, γδT cells categorize into four functionally distinct subpopulations: naïve γδT cells, central memory γδT cells, effector memory γδT cells, and terminally differentiated effector memory γδT cells (69). The latter two subpopulations express CD16, a surface receptor that efficiently facilitates tumor cell killing, even in the absence of antibody engagement (68, 70). Vγ9Vδ2 T cells, isolated from PBMCs of healthy donors, undergo activation, leading to the expression of CD16, a phenomenon not observed in their unstimulated counterparts (71). Furthermore, when TCR-activated Vγ9Vδ2 T cells are cross-linked to plastic wells with anti-CD16 monoclonal antibodies, substantial TNF-α production occurs, a response mitigated by the addition of soluble anti-CD16 monoclonal antibodies (71).

The cytotoxic activity of γδT cells is also ascribed to the expression of TRAIL and FasL, which bind to corresponding receptors on tumor cells (72, 73) (**Figure 1**). TRAIL’s interaction with different receptors produces varied outcomes: knockdown of TRAIL-Receptor 4(TRAIL-R4) in Colo357 and MDA-MB-231 cells significantly reduces sensitivity to γδT cell-induced cytotoxicity, whereas TRAIL-R4 knock-in HeLa cells show reinforced cytotoxicity (74). Furthermore, serum TRAIL levels hold clinical significance, as evidenced in a study involving eighteen patients with refractory prostate cancer, where higher serum TRAIL levels at nine months correlated with improved clinical outcomes (75). Additionally, the upregulation of Fas on the surface of osteosarcoma cells effectively increases the cytotoxicity of γδT cells (76).

Finally, γδT cells serves as potent producers of IFN-γ and TNF-α, exerting anti-tumor effects through various mechanisms, including the inhibition of tumor vascular growth (77) (**Figure 1**). Blocking TNF-α or its receptor significantly diminishes cytotoxicity, while knocking down miR-125b-5p could increase the secretion of IFN-γ and TNF-α, thereby enhancing anti-tumor effects (78, 79). Studies focusing on solid tumors, particularly breast cancer, nasopharyngeal carcinoma, and melanoma, have demonstrated a positive correlation between the production of TNF-α by peripheral γδT cells and their contribution to tumor defense (77).

### 2.2 Innate lymphoid cells (ILCs)

#### 2.2.1 NK cells

NK cells have consistently been acknowledged as effector cells proficient in lysing tumor cells or viruses, albeit with a non-specific targeting of cells. Upon recognizing target cells, NK cells exhibit directed movement of their abundant granules toward the binding site of target cells with the assistance of dynein motors (80, 81). The aggregation of these granules enhances efficiency in secretion while reducing the killing of surrounding cells. However, the cytotoxic impact of granules is contingent upon the presence of perforin. Mouse experiments have demonstrated that defective perforin leads to diminished cellular cytotoxicity, expedited tumor growth, and heightened metastasis, underscoring the crucial role of perforin in this process (82–84) (**Figure 1**). Currently, it is possible to induce the expression granzymes and perforin genes to augment the cytotoxic effects of NK cells.

Termed as “serial killers”, NK cells frequently shift towards cell destruction contingent upon FasL and TRAIL once their reservoirs of granzymes and perforin are depleted (85, 86) (**Figure 1**). Subsequent investigations have revealed NK cells deficient in perforin, previously considered lacking cytotoxicity, effectively eliminate MHC class I-deficient tumor cells due to the upregulation of FasL (87). FasL, in turn, interacts with the CD95 receptor on target cells, thereby initiating the apoptotic signaling cascade intrinsic to target cells (88). Intriguingly, the cleaved soluble form of FasL proves to be devoid of cytotoxic efficacy. Furthermore, NK cells harvested from the murine hepatic milieu distinctly express TRAIL, with their cytotoxicity potential markedly attenuated upon the introduction of anti-TRAIL monoclonal antibodies (85, 89).

Besides, IFN-γ secreted by NK cells has been demonstrated independently to exert anti-tumor functions, irrespective of perforin (**Figure 1**). Its collective influence plays a crucial role in governing the initiation, proliferation, and metastasis of tumors (90). Furthermore, while the specific anti-tumor mechanism of IFN-γ in particular tumors remains incompletely understood, its capabilities to inhibit tumor angiogenesis and modulate the sensitivity of tumor cells have long been reported (89).

In contrast, neoplastic cells may indeed serve as a crucial force driving the anti-tumor effects innitiated by NK cells (**Figure 1**). In melanoma, lactate derived from tumor cells significantly reduces the quantity and activation of NK cells. This is accomplished by suppressing the upregulation of the nuclear factor of activated T cells (NFAT) with NK cells, leading to a noticeable reduction in IFN-γ production and a simultaneous alleviation of the cytotoxic impact on tumors (91). In another study, it was revealed that lactate derived from tumor cells also directly diminishes the expression of perforin and granzyme, thereby impeding their cellular lytic functionality (92).

#### 2.2.2 Helper ILCs

ILCs earn their name due to their absence of adaptive antigen receptors. In addition to NK cells, other subsets include ILC1s, ILC2s, ILC3s, and lymphoid tissue inducer cells (LTi) (93). They predominantly inhabit tissues and maintain close associations with the extracellular matrix (93). Typically, within the tumor microenvironment, ILC1s release significant levels of IFN-γ. This cytokine acts on tumor cells, inducing the upregulation of MHC-I and MHC-II, thereby directly stimulating tumor cell apoptosis and pyroptosis (93). Moreover, both ILC1s and ILC3s possess the ability to directly eliminate tumor cells by expressing TRAIL, thereby imbuing these cells with the potential of anti-tumor effector cells (94) (**Figure 1**).

### 2.3 M1-type macrophages(M1 macrophages)

In the TME, a subset of infiltrating macrophages, referred to as tumor-associated macrophages (TAMs), exhibits the capacity to differentiate into two distinct polarization states: M1 macrophages and M2-type (M2) macrophages (95). The identification of new markers such as C-X-C motif chemokine ligand 9(CXCL9) and (secreted phosphoprotein 1)SPP1 challenges the conventional M1/M2 classification paradigm (96). CXCL9, produced by macrophages, plays a pivotal role in immune cell activation and signaling involved in inflammatory responses, thereby enhancing anti-tumor capabilities (97, 98). Conversely, SPP1 expressed in macrophages can boost the expression of interferon-gamma and interleukin-12, influencing macrophage polarization, migration, and cytokine profile (98). The CXCL9:SPP1 expression ratio holds greater clinical significance (98). These newfound markers present a nuanced perspective on the potential range of macrophage activation states, offering fresh insights and avenues for the advancement of targeted immunotherapy strategies. In the following discussion, we chose to describe the more traditional and extensively studied M1/M2 classical polarization.

M1 macrophages possess potent antimicrobial and anti-tumor activities, releasing cytotoxic molecules such as reactive oxygen species (ROS) and nitric oxide synthases (INOS), gradually causing damage to tumor cells (99–101) (**Figure 1**). In murine animal experiments, it has been demonstrated that M1 macrophages secrete these factors that delay the growth of ovarian cancer tumors (102). However, others argue that TAMs release nitric oxide (NO) and reactive oxygen intermediates (ROI), causing DNA damage and genetic instability in the initial stages, categorizing them as tumor-promoting factors (103). Another rapid method of cell destruction involves ADCC, as clearly shown by the vitamin D-dependent release of antimicrobial peptide cathelicidin. This peptide effectively targets the mitochondria of malignant cells, culminating in the demise of high-grade B-cell lymphoma entities (104). As previously found, TNF-α at the tumor site is primarily derived from M1 macrophages and tumor cells (105) (**Figure 1**). Early research demonstrated that exogenous TNF-α could promote the destruction of tumor vasculature, thereby indirectly leading to the necrosis of tumor cells (106). Subsequent studies showed that high levels of exogenous TNF-α administration may act directly on malignant cells by inducing apoptosis, although the specific mechanisms of this process are not yet fully understood (56).

### 2.4 Eosinophils

Despite their lower presence in the peripheral bloodstream compared to T cells or B cells, eosinophils are selectively recruited to the tumor microenvironment by chemotactic agents, such as high mobility group box one protein (HMGB1) (107). Subsequently, these granulocytes release a spectrum of mediators, causing a direct cytotoxic impact on tumor cells. The identified mediators encompass major basic protein (MBP), eosinophil cationic protein (ECP), and eosinophil peroxidase (EPX), all capable of inducing tumor cell lysis *in vitro (* 108, 109) (**Figure 1**). Furthermore, murine experiments focusing on colorectal cancer and lymphoma have revealed that the cytotoxic mediators wielded by eosinophils predominantly involve granule enzymes A and B (110–112).

In addition, in the presence of IL-5, eosinophils exhibit a significantly enhanced cytotoxic potency, coinciding with a noticeable deceleration in murine tumor growth (113). Moreover, when induced by lipopolysaccharide (LPS), eosinophils demonstrate the ability to directly undermine murine hepatic cancer cells via the release of TNF-α (114) (**Figure 1**). However, this phenomenon, can be effectively impeded by the administration of anti-TNF-α antibodies (115).

## 3 Direct tumor-promoting effects of immune cells and their counteractions

### 3.1 T cells

#### 3.1.1 CD4^+^ T cells

##### 3.1.1.1 Th2 cells

**Figure 2:**
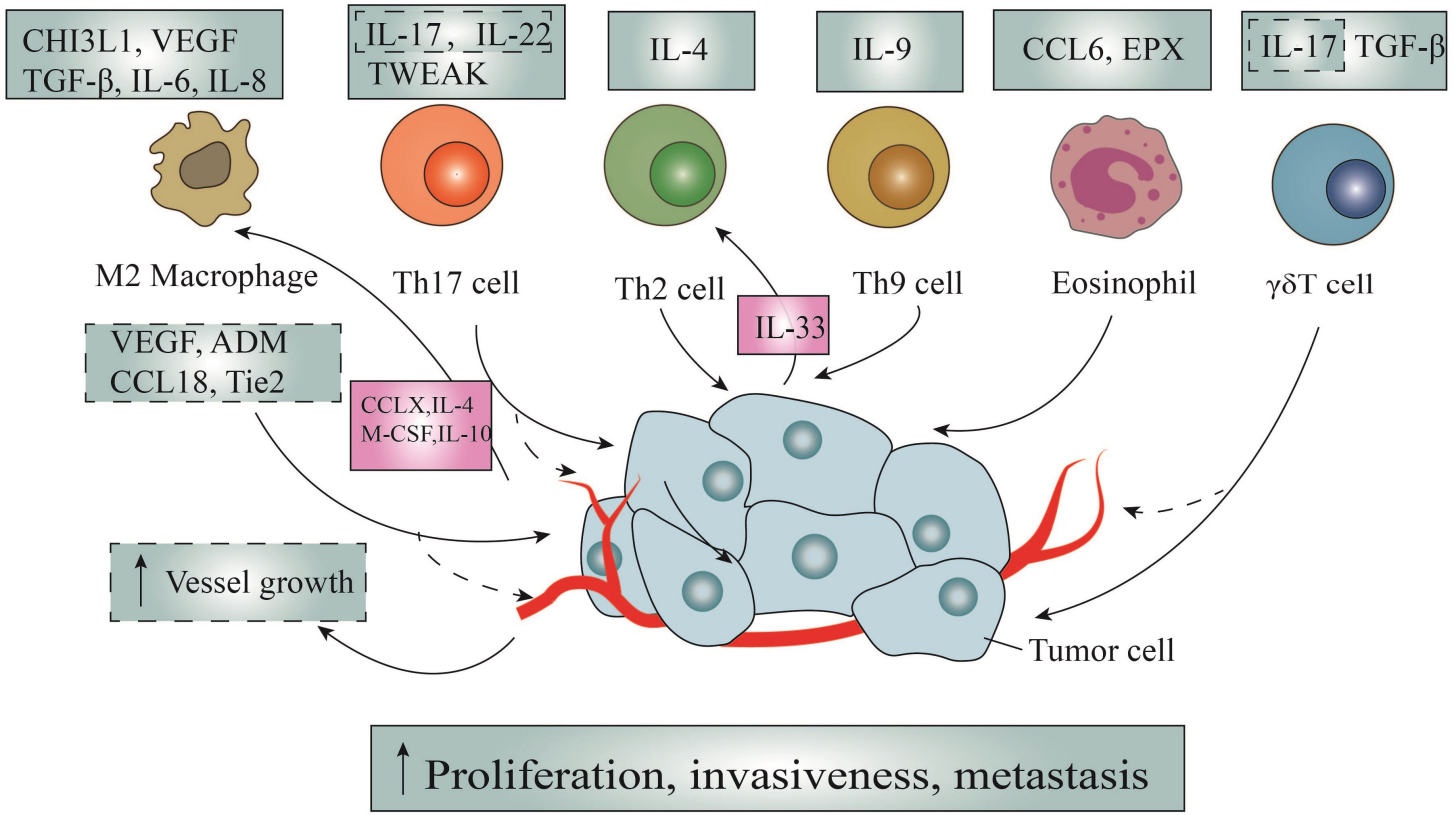
The direct tumor-promoting action of immune cells and the counteraction of tumor cells. Through the secretion of multiple chemokines, cytokines and other effector molecules such as IL-4, interleukin-5, and so on, immunocytes promote tumor cells through the following ways: promoting the proliferation of tumor cells, promoting the migration and metastasis of tumor cells and promoting tumor angiogenesis. It is worth noting that tumor cells can in turn promote the activation and recruitment of macrophages and Th2 cells via secreting CCLX, IL-33, IL-4, IL-10, and M-CSF, thus promoting the formation of loops.

The role of Th2 cells in allergic diseases has been extensively investigated, but their specific implications in tumor immunity remain elusive (116). Notably, several studies have highlighted a close association between Th2 cells in the TME and the progression and metastasis of breast, cervical, colorectal, and lung cancers (117). IL-4, a pivotal factor in Th2 cell polarization and a primary secretion of Th2 cells, is proposed as a potential mechanism for its direct impact on tumors (**Figure 2**). Firstly, in colorectal cancer, IL-4 induces the expression of epithelial-mesenchymal transition(EMT)-promoting proteins through signal transducer and activator of transcription 6(STAT6)-dependent transcription, thereby prompting EMT in colon cancer cells (118). Secondly, IL-4 stimulates the proliferation of pancreatic cancer cells by activating phosphorylation in mitogen-activated protein kinases(MAPK), Akt-1, STAT3, and insulin receptors (119). *In vitro* experiments have additionally demonstrated that IL-4 promotes the expression of anti-apoptotic genes in various human cancers (120).

Besides, recent research has also highlighted the interplay between tumor-infiltrating Th2 cells and tumor cells, where tumor fungal elements activate signaling pathways in cancer cells, promoting the secretion of IL-33, which is essential for the recruitment and activation of Th2 cells (121) (**Figure 2**). Conversely, the genetic deletion of IL-33 or antifungal therapy results in the regression of stable pancreatic ductal adenocarcinoma (PDAC), underscoring the tumor-promoting nature of Th2 cells. Despite this, concrete evidence substantiating the tumor-promoting effects of Th2 cells remains limited (121).

##### 3.1.1.2 Th9 cells

Tumor-infiltrating Th9 lymphocytes release the characteristic cytokine IL-9, which has been implicated in various immune and inflammatory diseases, including parasitic infections, allergies, and lymphoma (122). However, the precise and consistent role of IL-9 in tumor immunity remains enigmatic and subject to controversy (**Figure 2**). According to existing literature, IL-9 binds to heterodimer receptors, activating the Janus kinase(JAK)-STAT, insulin receptor substrates(IRS), and MAPK signaling pathways, thereby directly stimulating tumor cell proliferation (123). Additionally, investigations have indicated that overexpression of IL-9 leads to amplified proliferation of colonic epithelial cells, attributed to the upregulation of c-MYC and cyclin D1 expression (124).

EMT, a pivotal mechanism underlying tumor metastasis, involves profound phenotypic alterations such as cytoskeletal reorganization, detachment from the extracellular matrix, and loss of polarity (125). Salazar et al. conducted a study encompassing lymphocyte co-cultures, *in vivo* mouse models, and human lung cancer tissues (126). The study revealed that tumor-infiltrating Th9 cells induce EMT and migration, and metastatic expansion of lung cancer. Similarly, others disclosed that IL-9 exerts notable influence on increasing the expression of C-C chemokine ligand 20 (CCL20) in hepatocellular carcinoma cells, thereby eliciting EMT changes through STAT3 phosphorylation (127).

##### 3.1.1.3 Th17 cells

Named after their hallmark product, IL-17A, Th17 cells are considered a major component among infiltrating tumor lymphocytes, concurrently secreting IL-17F, IL-21, IL-22, and IL-2 (128). First, IL-17, originating from Th17 cells, serves as a stimulant for tumor cell proliferation across diverse pathways (129) (**Figure 2**). For instance, B-cell acute lymphoblastic leukemia relies on Akt and STAT3 pathways, colorectal cancer involves STAT3 and NF-*κ*B pathways, and ovarian cancer stem cells necessitate the engagement of NF-*κ*B and MAPK pathways (130, 131). In a recent investigation, it has been unveiled that the secretion of tumor necrosis factor-like weak inducer of apoptosis(TWEAK) by Th17 cells triggers epithelial-mesenchymal transition, consequently fostering liver metastasis in colorectal cancer (132) (**Figure 2**).

Moreover, within the domain of lung cancer research, the interaction between Th17/Treg cells and their impact on non-squamous non-small cell lung cancer (NSCLC) biology has garnered attention. These emphasize that Th17 cells not only induce EMT in lung cancer cells, but also augment migration and dissemination, correlating with lymphatic vessel density (126). Subsequent investigations have provided evidence linking IL-17 and IL-22 to increased invasiveness and metastasis of lung cancer cells (**Figure 2**). Furthermore, these studies have demonstrated resistance to combined MEK inhibitor and anti-PD-L1 therapies in KRAS/p53 mutant lung cancers (133). In the context of non-small cell lung cancer, IL-17A actively promotes migration and invasion through the STAT3/NF-*κ*B/Notch1 signaling pathway (134). Despite the substantial roles of IL-17 and IL-22 in inducing angiogenesis, facilitating EMT, and expressing matrix metalloproteinases (MMPs) to promote tumor growth and tumor metastasis, there is currently a dearth of literature specifying the specific sources of these two cytokines (135–139).

#### 3.1.2 γδT cells

While traditionally recognized for their potent anti-tumor effects, γδT lymphocytes also possess the potential to accelerate the progression and invasive tendencies of solid tumors (140). Nonetheless, compelling evidence suggests that γδT cells may expedite the development and invasion of solid tumors (141). At the core of their tumorigenic impact is the pivotal mediator IL-17, a molecule that not only drives neoplastic cell proliferation through intricate IL-6/STAT3 and NF-*κ*B signaling cascades but also triggers metastasis by inducing the secretion of VEGF and MMP (142). Furthermore, under specific circumstances, epithelial Vδ1 T cells have been observed to secrete notable quantities of TGF-β, initiating the transformation of epithelial cells into mesenchymal cells and thereby amplifying the invasiveness of malignancies (143) (**Figure 2**).

### 3.2 M2 macrophages

In contrast to the anti-tumor effects associated with M1 macrophages discussed earlier, M2 macrophages are typically considered closely associated with promoting tumor metastasis (**Figure 2**). Notably, macrophage-colony stimulating factor(CSF-1), primarily sourced from macrophages, has been found to be correlated with poor prognosis in breast cancer, ovarian cancer, endometrial cancer, lung cancer, and prostate cancer, though the detailed underlying mechanisms remain unclear (144, 145). M2 macrophages promote metastasis by producing MMPs and tissue proteases, which degrade the extracellular matrix, allowing invasive tumor cells to migrate into surrounding tissues and the vascular system (145). Secondly, M2 macrophages can promote lymph node metastasis of tumor cells by enhancing the functionality of lymphatic vessels. Additionally, M2 macrophages play roles in inducing the formation of tip cells in lymphatic endothelial cells (LECs) and the proliferation of lymphocytes through the secretion of VEGF-C and the expression of podoplanin (146).

The promotion of tumor metastasis by M2 macrophages is closely associated with the formation of new tumor blood vessel as well (147, 148) (**Figure 2**). The MMP9 produced by these macrophages typically facilitate the release of VEGF from the extracellular reservoir, thereby increasing the bioavailability of VEGF (147). Although TAM infiltration is predominantly associated with extensive angiogenesis via VEGF signaling pathway, studies have shown that disrupting the VEGFA allele effectively impacts vascular sprouting without affecting the recruitment of macrophages and angiogenesis. Further research has demonstrated that this is closely associated with TAM-derived adrenomedullin (ADM) and C-C motif ligand 18(CCL18) (149, 150). Respectively ADM promotes angiogenesis and melanoma growth via the paracrine effect, mediated by the endothelial nitric oxide synthase signaling pathway, and CCL18 promotes human umbilical vein endothelial cell migration and tube formation via PITPNM3 (149, 150). Additionally, the expression of the Tie2 receptor by these macrophages is a known receptor for angiopoietin, playing a crucial role in angiogenesis. Additionally, the expression of the Tie2 receptor by these macrophages is a known receptor for angiopoietin, playing a crucial role in angiogenesis (151).

The EMT is a process in which epithelial cells gradually lose their epithelial characteristics and acquire a mesenchymal phenotype, playing a crucial role in tumor cell metastasis. Macrophages exhibit high infiltration in the tumor microenvironment, secreting a series of inflammatory and cytokine factors to promote EMT and enhance the stemness of cancer cells (152) (**Figure 2**). For instance, IL-6 derived from M2 macrophages has been found to downregulate the epithelial marker E-cadherin and upregulate the mesenchymal marker vimentin in cancer cells (145). Additionally, M2 macrophages can also secrete TGF-β to induce Sox9 expression in lung cancer cells through the c-Jun/Smad3 pathway, thereby inducing EMT and enhancing lung cancer cell migration (145). IL-8 also has the ability to induce EMT by activating the JAK2/STAT3/Snail pathway (153). Moreover, TAMs regulate breast cancer stem cell phenotype and promote tumor growth via the EGFR/Stat3/Sox-2 signaling pathway (154).

Several other cytokines derived from M2 macrophages also play vital roles, as follows (**Figure 2**). For instance, IL-6 has been shown to activate cancer stem cells, facilitating cancer growth and metastasis by promoting anti-apoptotic pathways through STAT3 phosphorylation (155, 156). As we all know, Chitinase 3-like protein -1 (CHI3L1), as a glycoprotein, assumes a pivotal role in governing various aspects of tumor cell behavior, including growth, proliferation, invasion, metastasis, angiogenesis, and activation (157). Correspondingly, CHI3L1, derived from M2 macrophages in mice, facilitates the metastasis of gastric cancer and breast cancer through the IL-13 receptor (158).

Surprisingly, tumor cells often react against TAMs in a way that amplifies their facilitation (**Figure 2**). TAMs originate from peripheral monocytes, recruited into tumors by several growth factors, particularly those produced by matrix and tumor cells (159). Macrophages’ polarization is regulated by various microenvironmental signals from tumor cells, such as IL-4 and IL-10, which serve the same purpose (160). In addition to macrophage colony-stimulating factor (M-CSF) and tumor-derived factors such as chemokines CCL2, CCL3, CCL4, and CCL5, which serve as macrophage chemoattractants, CCL2 is extensively expressed in various human tumors (161). For example, cancer cells produce CCL2 to recruit inflammatory CC chemokine receptor 2(CCR2) monocytes from blood to metastatic sites, where they differentiate into related macrophages and promote tumor cell extravasation under the influence of VEGF (162).

### 3.3 Eosinophils

Initially, eosinophils were commonly associated with specific inflammatory issues, particularly allergies and parasitic infections (114). However, as our knowledge grows regarding how inflammatory factors play a role in starting and advancing tumor cells, there has been a recent reevaluation of the role of eosinophils in this process. Preliminary studies have confirmed MMP9’s role in extracellular matrix degradation (163). Still, we are not completely sure about how this contributes to tumor cell invasion and spreading. Likewise, in situations with inflammation, MBP has been seen to make blood vessel cells multiply and boost the growth effects of VEGF (164). However, we are still working to confirm its similar role in the tumor environment (164).

Moving forward, substantial progress has been achieved in investigating eosinophils in solid tumors. According to Vasilios and his team, EPX from eosinophils has been observed to encourage tumor spreading in a mouse breast cancer model using the 4T1 strain (165). Furthermore, eosinophils have been strongly linked to speeding up the movement and spread of tumor cells in melanoma, credited to the release of a substance called CCL6 (166) (**Figure 2**).

## 4 Therapeutic strategies according to the mechanisms of direct effects of immunocytes on cancer cells

Despite atechnological advances, immunotherapy and targeted therapy remain key cancer treatments. Common immune checkpoints, exemplified by programmed death 1(PD-1), upon binding with programmed cell death-ligand 1(PDL-1), are typically expressed on the surface of tumor cells, orchestrating the inhibition of T cell proliferation and activation, thereby facilitating the evasion of tumor cells from immune surveillance (167). A parallel player in this regulatory milieu is cytotoxic T lymphocyte-associated antigen-4(CTLA-4), predominantly curtailing T cell activation and proliferation through competitive interference with the engagement of CD28 and co-stimulatory molecules CD80/86 (168). Additionally, lymphocyte activation gene-3(LAG-3) and T-cell immunoglobulin and mucin-domain containing-3(TIM-3) serve as pivotal suppressors of T cell activation and functionality by respectively engaging with MHC-II molecules and the ligand Galectin-9 (169). These two are typically not expressed on tumor cells, but are mainly expressed on T cells. Presently, PD-1 inhibitors such as Pembrolizumab and Nivolumab, along with PDL-1 inhibitors like Atezolizumab and Avelumab, as well as the CTLA-4 inhibitor Ipilimumab, stand as stalwarts in clinical intervention (170). Meanwhile, agents targeting LAG-3, TIM-3, among others, represented by BMS-986016 and MBG453, traverse the clinical research terrain, poised to offer therapeutic avenues for diverse malignancies in the forthcoming era. Nevertheless, notwithstanding the therapeutic promise, the response rate to immune checkpoint therapy remains modest, with resistance posing a formidable challenge (171, 172). Since current methods primarily enhance indirect anti-tumor effects, there is a pressing need to understand and explore direct anti-tumor therapies for comprehensive.

In this review, we focus mainly on the direct anti-tumor mechanisms of immune cells from three perspectives: amplifying the ADCC effect, triggering the secretion of granzymes and perforin, and modulating the Fas/FasL pathway (**Table 1**). Firstly, monoclonal antibodies such as Rituximab(anti CD20) and Trastuzumab(anti-HER2) have shown effectiveness via the ADCC pathway (173). Noteworthy studies involving mice lacking the Fcγ chain have revealed increased ADCC-mediated cytotoxicity in the absence of Fc gammaRIIB, while optimal antibody binding minimizes inhibitory effects via Fc gammaRIIB (174). Other antibodies, such as Cetuximab(anti-EGFR), Pertuzumab(anti-HER2), and bispecific antibodies-Catumaxomab, have opened new avenues in clinical research for gastrointestinal and breast cancer treatments (175). In addition, clinical drugs like Anktiva and Nemvaleukin alfa (ALKS 4230) stimulate the secretion of cytokines, such as IL-2, IL-21, and IL-15, to enhance the ADCC effect of NK cells (176). In the most recent study, researchers have devised a high-affinity, non-cleavable CD16 variant. Upon fusion with the NK cell activation domain, this novel construct robustly augments anti-tumor cell activity via the ADCC pathway (177). This approach primarily focuses on NK cells, which is less common in research involving other immune cells (178, 179).

**Table 1:** The effects of immune cells on tumor cells and their related mechanisms in the process.

Immune cells	Mechanisms	Biology effects	Refs
Th1 cell	INF-γ, TNF-α	Induces tumor senescence and apoptosis	35-38
Th2 cell	IL-4	Promots tumor proliferation and inhibits the apoptosis	118-120
Th9 cell	Granzyme	Induces poptosis in tumor cells	23, 40-41
	IL-9/IL-9R	Induces tumor cell cycle arrest and apoptosis	42-44
	IL-9/IL-9R	Promotes tumor growth and metastasis	123-124, 127
Th17 cell	IL-17	Promotes tumor proliferation, migration, and invasion	126, 129-131, 133-134
	TWEAK	Promotes cellular epithelial-mesenchymal transition.	132
γδT cell	Fas-Fasl	Induces apoptosis of tumor cells	76
	Granzyme	Induces apoptosis of tumor cells	64-67
	ADCC	Induces apoptosis of tumor cells	68-71
	TRAIL	Induces apoptosis of tumor cells	72-75
	INF-γ, TNF-α	Inhibits the growth of tumor vascular	77-79
	IL-17	Promotes tumor cell proliferation and metastasis	142
	TGF-β	Promotes the tumor invasiveness	143
CTL	Granzyme	Induces apoptosis of tumor cells	46-51
	Fas-Fasl	Induces apoptosis of tumor cells	52-53
	INF-γ, TNF-α	Influences the metabolism of tumor cells and promotes the rupture of tumor blood vessels	54-57
NK cell	Granzyme	Induces apoptosis of tumor cells	80-84
	TRAIL	Induces apoptosis of tumor cells	85, 89
	Fas-Fasl	Induces apoptosis of tumor cells	87-88
	INF-γ	Inhibits the growth of tumor vascular and changes the sensitivity of tumor cells	90
Helper ILC	INF-γ	Stimulating tumor cell apoptosis and pyroptosis	93
	TRAIL	Induces apoptosis of tumor cells	94
Macrophage2	VEGF	Promotes migration and invasion	147-148
	ADM, CCL18, Tie2	Promotes the generation of tumor blood vessels	149-151
	IL-6, IL-8, CHI3L1	Promotes growth and migration	145, 152-153, 155-158
Eosinophil	MBP, ECP, EPX	Induce lysis of tumor cells	108, 109
	Granzyme	Induces apoptosis in tumor cells	110-112
	TNF-α	Induces apoptosis of tumor cells	114-115
	MBP, EPX, CCL6	Promotes tumor metastasis	163-164, 166
Macrophage1	ROS, INOS	Induces apoptosis of tumor cells	100-101
	TNF-α	Promotes the destruction of tumor vasculature	56, 106
	ADCC	Induces apoptosis of tumor cells	104

Chimeric antigen receptor-modified T(CAR-T) technology enhances the release of perforin and granzymes, transforming CTLs, Th cells, NK cells, and other cells into powerful weapons for eliminating tumor cells (180). Additionally, the NKp30 receptor serves as another specific receptor for CAR-T technology, triggering the secretion of granzymes and perforin upon when binding to B7-H6l (181). However, there is a lack of developed antibodies or small ligands targeting NKp30. Despite various studies demonstrating the presence of perforin and granzyme B in T cells from CAR patients, resulting the cleavage of fibronectin extra domain B-positive cells and the induction of apoptosis, effective therapeutic interventions are still pending (158, 182, 183). In addition, blocking immune checkpoints can alleviate the suppression of the expression of perforin and granzymes, enhancing cytotoxicity (184). Furthermore, in *in vitro* experiments, it has been demonstrated that the use of PD-1 blockade drugs can effectively boost the cytotoxicity of γδ T cells (185). Furthermore, *in vitro*, assays revealed that either Bacillus Calmette-Guéri or Zoledronate treatment of bladder tumor cells induced granzymes (186). Ongoing experiments are focused on investigating fluorescent biosensors, allowing for a more specific and sensitive assessment of granzyme B activity (187).

Compared to the involvement of granzyme and perforin, the Fas/FasL pathway and secreted cytokines, as another potent anti-tumor target, can significantly and directly enhance the tumor-killing efficacy (188). Traditional chemotherapy drugs like Doxorubicin and Methotrexate induce DNA damage in immune cells, leading to the expression of FasL on their surface to bolster the effectiveness of the immune system (189, 190). Undoubtedly, the application of antibodies, such as R-125224, is undeniable in this context. Moreover, FasL gene therapy is also actively under development, though its practical implementation remains contentious. Common delivery methods encompass adenovirus delivery, FasL-engineered cell delivery, and attenuated bacterial delivery (191). Despite, IFN-γ being an FDA-approved drug for treating chronic granulomatous disease and osteopetrosis, its approval for malignancy treatment is currently pending (192).

Given the evolving landscape of direct tumor-modulating mechanisms of immune cells, strategies to curtail tumor cell proliferation and inhibit blood vessel growth have garnered exploration (**Table 1**). Despite their crucial role in regulating tumor cell proliferation, differentiation, and apoptosis (193), the precise mechanisms and long-term consequences of STAT3 and STAT5 remain relatively unknown (194). Among the few inhibitors targeting the SH2 domain of STAT3 and interacting with STAT5, OPB-31121 has shown anti-tumor activity in leukemia, with ongoing phase I/II clinical trials assessing efficacy against solid tumors and hematopoietic cancers (195).

The prominence of EMT in tumor progression has galvanized extensive research into approaches for tumor treatment (196, 197) (**Table 1**). In a recent investigation, Soundararajan et al. embarked on exploring the potential of combining EMT therapy to overcome resistance to immunotherapy, presenting a promising strategy for enhancing treatment outcomes (198, 199). Targeting upstream pathways of EMT can significantly inhibit tumor growth, with TGF-β signaling being the most prominent inducer of EMT (200). Extensive research has focused on evaluating the effectiveness of TGF-β inhibitors, such as LY2157299, as potent anti-EMT compounds in ongoing clinical trials (201, 202). Similarly, targeting upstream transcription factors of EMT has been proposed as a feasible therapeutic alternative for invasive cancers (203–206). Furthermore, another treatment option for EMT-dependent cancers is targeting the stromal cells, with an exciting approach being to target the stromal cells themselves by inhibiting stroma-specific proteins with monoclonal antibodies (207). This has been validated in a mouse model of breast cancer (208). However, the current therapeutic approaches for EMT programs remain rudimentary, suggesting an exciting avenue for future developments in highly effective therapies to manage high-grade tumor malignancies.

In addition to the aforementioned factors, MMPs are other major mediators for metastasis and invasion of tumor cells in the tumor microenvironment (209) (**Table 1**). Though attempts to develop drugs targeting MMPs were made twenty-five years ago, phase III clinical trials evaluating small molecule metalloproteinase inhibitors (MPIs) yielded disappointing, failing to improve survival rates for cancer patients. The limited efficacy of MPIs for palliative care has been widely recognized (201). Currently, MMP inhibitory monoclonal antibodies are considered promising MMP-targeted therapies, as they offer higher target selectivity and better pharmacokinetic properties compared to small molecule drugs (210). Inhibitory monoclonal antibodies targeting individual MMP-9 and MMP-14 have been developed and demonstrated anti-tumor activity in preclinical models of breast cancer, which could become a promising area of research in the future (211–214).

Ultimately, interfering with tumor vasculature has emerged as a promising strategy to inhibit tumor growth (202, 215, 216) (**Table 1**). Notably, Bevacizumab(anti-VEGF), an FDA-approved drug for previously untreated metastatic colorectal cancer, has demonstrated remarkable effects, extending its application to diverse malignant tumors, including NSCLC, renal cell carcinoma, ovarian cancer, and cervical cancer (215, 217). Another fusion protein capable of effectively targeting angiogenesis by inhibiting VEGF-A, VEGF-B, and placental growth factor is Ziv-aflibercept, which has also been brought to market. It is worth noting that, compared to bevacizumab, it exhibits a higher binding affinity to VEGF-A (218, 219). Additionally, Ramucirumab is a human IgG1 monoclonal antibody that acts as an inhibitor of VEGFR2 (220). It works by binding to and inhibiting the activation of VEGFR2, thereby suppressing the signaling pathways mediated by VEGF (220). other drugs like Aflibercept are currently under development, showing promising potential in inhibiting tumor vasculature (221).

## 5 Conclusions

Despite significant progress in cancer treatment, the ongoing existence of malignant tumors highlights persistent challenges such as immune suppression, evasion, and tolerance. Given the pivotal role of immune cells within the TME, this review comprehensively delves into the direct, intricate, and bidirectional impacts they exert on tumor cells. These dynamic interactions unveil a complex pattern, wherein distinct immune cell cohorts may paradoxically propel tumor progression or incite robust antitumor responses across varied tumor microenvironments. Precision interventions aimed at enhancing immune cell cytotoxicity or diminishing their tumor-promoting effects show promise in overcoming the challenges presented by the dual nature of immune cells and the intricate landscape of indirect immune regulation.

However, in the overall scheme, the efficacy of tumor treatment is closely related to the immune environment of tumor patients, going beyond just describing the direct interactions between immune cells and tumor cells as outlined in this paper. The indirect influences of immune cells, including the regulation of T cells and fibroblasts, need to be considered. Additionally, the emergence of novel immune cell markers may indicate the emergence of diverse subgroups of immune cells with various functionalities and contributions to tumor biology. These new insights challenge traditional paradigms of immune polarization, emphasizing the importance of a detailed understanding of immune cell heterogeneity in oncology and highlighting the complex composition of immune cell biology. Therefore, exploring the intricately complex components of the tumor microenvironment, understanding their specific, direct mechanisms of action, can yield valuable insights into slowing tumor progression, controlling drug resistance, and more.

## Author contributions

ZY: Writing – original draft, Writing – review & editing. PC: Writing – original draft, Writing – review & editing. QH: Writing – original draft, Writing – review & editing. JH: Writing – original draft. LH: Conceptualization, Writing – review & editing. GH: Conceptualization, Funding acquisition, Resources, Writing – review & editing.

## Glossary

FAS: Factor associated suicide
FASL: Factor associated suicide ligand
TRAIL: Tumor necrosis associated apoptosis-inducing ligand
HER2: Human epidermal growth factor receptor 2
TNF: Tumor necrosis factor
INK4a: The inhibitor of cyclin-dependent kinase 4a
Blimp-1: B-lymphocyte-induced maturation protein 1
VEGF-A: Vascular endothelial growth factor-A
PGE2: Prostaglandin E2
MHC: Major histocompatibility complex
PBMCs: Peripheral blood mononuclear
IPP: Isopentenyl pyrophosphate
SCCHN: Squamous cell carcinoma of the head and neck
CMA: Concanamycin A
ADCC: Antibody-dependent cell-mediated cytotoxicity
ILCs: Innate lymphoid cells
NFAT: Nuclear factor of activated T cells
LTi: Lymphoid tissue inducer cells
TAMs: Tumor-associated macrophages
M1 macrophages: M1-type macrophages
M2 macrophages: M2-type macrophages
ROS: Reactive oxygen species
iNOS: Nitric oxide synthases
NO: Nitric oxide
ROI: Reactive oxygen intermediates
HMGB1: High mobility group box one protein
MBP: Major basic protein
ECP: Eosinophil cationic protein
EPX: Eosinophil peroxidase
LPS: Lipopolysaccharide
EMT: Epithelial-mesenchymal transition
STAT6: Signal Transducer and Activator of Transcription 6
MAPK: Mitogen-activated protein kinases
PDAC: Pancreatic ductal adenocarcinoma
IRS: Insulin receptor substrates
JAK: Janus kinase
CCL20: C-C chemokine ligand 20
NSCLC: Non-squamous non-small cell lung cancer
MMPs: Expressing matrix metalloproteinases
CSF-1: Macrophage-colony stimulating factor
LECs: Lymphatic endothelial cells
ADM: TAM-derived adrenomedullin
CHI3L1: Chitinase 3-like protein -1
M-CSF: Macrophage colony-stimulating factor
CCR2: CC chemokine receptor 2
PD-1: Programmed death 1
CTLA-4: Cytotoxic T lymphocyte-associated antigen-4
PDL-1: Programmed cell death-ligand 1
LAG-3: Lymphocyte activation gene-3
TIM-3: T-cell immunoglobulin and mucin-domain containing-3
CAR-T: Chimeric antigen receptor-modified T
MPIs: Metalloproteinase inhibitors
CXCL9: C-X-C motif chemokine ligand 9
SPP1: secreted phosphoprotein 1
TWEAK: tumor necrosis factor-like weak inducer of apoptosis
